# Directed Evaluation of Enterotoxigenic *Escherichia coli* Autotransporter Proteins as Putative Vaccine Candidates

**DOI:** 10.1371/journal.pntd.0001428

**Published:** 2011-12-06

**Authors:** Jessica A. Harris, Koushik Roy, Virginia Woo-Rasberry, David J. Hamilton, Rita Kansal, Firdausi Qadri, James M. Fleckenstein

**Affiliations:** 1 University of Tennessee College of Medicine, Memphis, Tennessee, United States of America; 2 Department of Medicine, University of Tennessee Health Sciences Center, Memphis, Tennessee, United States of America; 3 Research Services, Veterans Affairs Medical Center, Memphis, Tennessee, United States of America; 4 Department of Comparative Medicine, University of Tennessee Health Sciences Center, Memphis, Tennessee, United States of America; 5 International Centre for Diarrheal Disease Research, Dhaka, Bangladesh; 6 Department of Molecular Sciences, University of Tennessee Health Sciences Center, Memphis, Tennessee, United States of America; 7 Department of Medicine, Veterans Affairs Medical Center, Memphis, Tennessee, United States of America; Instituto Butantan, Brazil

## Abstract

**Background:**

Enterotoxigenic *Escherichia coli* (ETEC) is a major diarrheal pathogen in developing countries, where it accounts for millions of infections and hundreds of thousands of deaths annually. While vaccine development to prevent diarrheal illness due to ETEC is feasible, extensive effort is needed to identify conserved antigenic targets. Pathogenic *Escherichia coli*, including ETEC, use the autotransporter (AT) secretion mechanism to export virulence factors. AT proteins are comprised of a highly conserved carboxy terminal outer membrane beta barrel and a surface-exposed amino terminal passenger domain. Recent immunoproteomic studies suggesting that multiple autotransporter passenger domains are recognized during ETEC infection prompted the present studies.

**Methodology:**

Available ETEC genomes were examined to identify AT coding sequences present in pathogenic isolates, but not in the commensal *E. coli* HS strain. Passenger domains of the corresponding autotransporters were cloned and expressed as recombinant antigens, and the immune response to these proteins was then examined using convalescent sera from patients and experimentally infected mice.

**Principal Findings:**

Potential AT genes shared by ETEC strains, but absent in the *E. coli* commensal HS strain were identified. Recombinant passenger domains derived from autotransporters, including Ag43 and an AT designated pAT, were recognized by antibodies from mice following intestinal challenge with H10407, and both Ag43 and pAT were identified on the surface of ETEC by flow cytometry. Likewise, convalescent sera from patients with ETEC diarrhea recognized Ag43 and pAT, suggesting that these proteins are expressed during both experimental and naturally occurring ETEC infections and that they are immunogenic. Vaccination of mice with recombinant passenger domains from either pAT or Ag43 afforded protection against intestinal colonization with ETEC.

**Conclusions:**

Passenger domains of conserved autotransporter proteins could contribute to protective immune responses that develop following infection with ETEC, and these antigens consequently represent potential targets to explore in vaccine development.

## Author Summary

Diarrheal diseases are responsible for more than 1.5 million deaths annually in developing countries. Enterotoxigenic *E. coli* (ETEC) are among the most common bacterial causes of diarrhea, accounting for an estimated 300,000–500,000 deaths each year, mostly in young children. There unfortunately is not yet a vaccine that can offer sustained, broad-based protection against ETEC. While most vaccine development effort has focused on plasmid-encoded finger-like ETEC adhesin structures known as colonization factors, additional effort is needed to identify conserved target antigens. Epidemiologic studies suggest that immune responses to uncharacterized, chromosomally encoded antigens could contribute to protection resulting from repeated infections. Earlier studies of immune responses to ETEC infection had identified a class of surface-expressed molecules known as autotransporters (AT). Therefore, available ETEC genome sequences were examined to identify conserved ETEC autotransporters not shared by the commensal *E. coli* HS strain, followed by studies of the immune response to these antigens, and tests of their utility as vaccine components. Two chromosomally encoded ATs, identified in ETEC, but not in HS, were found to be immunogenic and protective in an animal model, suggesting that conserved AT molecules contribute to protective immune responses that follow natural ETEC infection and offering new potential targets for vaccines.

## Introduction

Enterotoxigenic *Escherichia coli* (ETEC) are a major cause of diarrheal illness in developing countries where these organisms cause hundreds of millions of infections and an estimated 300,000–500,000 deaths in young children each year [1]. ETEC are perennially by far the most common cause of traveler's diarrhea [2]. Disease caused by ETEC is highly endemic in regions plagued by inadequate sanitation and a lack of clean drinking water, and prevention of ETEC is a high priority [1], [3]. ETEC are genetically heterogeneous pathogens that share the ability to colonize the small intestine where they deliver the cholera toxin-like heat-labile toxin (LT) and/or small peptide heat-stable (ST) toxins that ultimately result in diarrhea [4].

In the classic paradigm for ETEC pathogenesis, small intestinal colonization requires plasmid-encoded colonization factors (CFs) [4]. A variety of more than 25 antigenically distinct fimbrial, or fibrillar CFs have been described to date [5], [6]. These antigens, along with LT, remain central to ETEC vaccine development [7]. However, CF antigens are not appreciably cross-protective, and many ETEC strains do not appear to produce CFs [8], [9]. Moreover, LT alone (or the homologous cholera toxin) do not appear to afford complete sustained protection [10], while ST, typically only 19 amino acids in its mature form, is not suitably immunogenic.

These constraints, as well as a growing appreciation of the complexity of ETEC pathogenesis [4], [11], have prompted searches for additional surface-expressed antigens. Use of classical genetic approaches including Tn*phoA* mutagenesis to find novel molecules exposed on the surface of ETEC, recently led to the identification of several putative virulence loci, including the etpBAC two-partner secretion locus [12], responsible for secretion of the EtpA adhesin molecule [13], and the autotransporter (AT) protein EatA [14].

EatA and other AT proteins contain three essential domains: an amino terminal signal peptide, the secreted “passenger” domain, and a third carboxy-terminal beta barrel domain inserted into the outer membrane [15]. The variable passenger portion of the protein may be cleaved by surface proteases and freely secreted as in the case of EatA, or remain attached to the transport domain. The surface expression of AT passenger domains proteins make them attractive targets for vaccine development, while only limited portions of the beta regions are predicted to be exposed [16].

While a broadly protective ETEC vaccine remains outstanding, one approach currently being explored is a protein subunit vaccine based on multiple ETEC antigens. Present acellular pertussis vaccines [17], [18], [19], subunit formulations containing a two-partner secretion (TPS) exoprotein adhesin (filamentous hemagglutinin, FHA) [20], [21], the pertactin autotransporter [22], [23], and pertussis toxoid offer a potential strategy that might be adopted to guide ETEC vaccine development. Indeed, recent investigations of EtpA [12], [13], [24], [25], an ETEC TPS exoprotein adhesin, were prompted by its similarity to FHA. Recent immunoproteomic studies of ETEC H10407 independently identified EtpA as well as several AT proteins including EatA, TibA and antigen 43 suggesting that these proteins are expressed during both experimental infection in mice and in humans [26].

Interestingly, it appears clear that children repeatedly exposed to ETEC infections are ultimately protected against subsequent symptomatic infections [27]. However, the precise composition of the protective antigens remains uncertain [4], [28], [29]. The present studies were performed to examine the possible contribution of conserved, chromosomally-encoded AT proteins to protective ETEC immune responses, and to evaluate passenger domains of these ATs as possible candidates for ETEC vaccine development.

## Methods

### Bacterial strains and plasmids

A complete list of strains and plasmids employed in these studies is included in table 1. ETEC strains H10407 and E24377A were originally provided by Marcia Wolf and Stephen Savarino, respectively, from cGMP lots maintained at Walter Reed Army Institute of Research.

**Table 1:** Bacterial strains and plasmids.

strains		
strain	description	source/reference(s):
enterotoxigenic E. coli strains		
B7A	ETEC strain^b^. origin: Vietnam; serotype O148∶H28; CS6; LT+/ST+; EtpA−	[69], [70]
H10407	ETEC strain^a^. origin: Bangladesh; serotype O78∶H11;CFA/1;LT+/ST+;EtpA+	[71], [72]
E24377A	ETEC strain^b^. origin: Egypt; serotype O139∶H28;CFA/II (CS1/CS3);LT+/ST+; EtpA+	[73]
Jf876	ΔlacZYA::Km^R^ derivative of H10407	[40]
recombinant E. coli strains		
DH5α	F- ϕ80*lac*ZΔM15 Δ(l*ac*ZYA-*arg*F) U169 *rec*A1 *end*A1 *hsd*R17 (r_k_−, m_k_+) *pho*A *sup*E44 λ- thi-1 *gyr*A96 *rel*A1	Invitrogen
BL21-A1	F- *ompT hsd*SВ (rB-mB-) *gal dcm ara*B::*T7RNAP*-*tet*A	Invitrogen
*ccd*B survival	F- *mcrA* Δ(*mrr-hsd*RMS-*mcr*BC) Φ80*lac*ZΔM15 Δ*lac*X74 *rec*A1 *ara*Δ139 Δ(*ara-leu*)7697 *gal*U *gal*K *rps*L (StrR) *end*A1 *nup*G	Invitrogen

plasmids		
plasmid	description	
pDONR221	4762 bp *att*P1/*att*P2 sites flanking CmR and *ccd*B; KmR	Invitrogen
pDEST17	6354 bp T7-6His-*att*R1-CmR-*ccd*B-*att*R2/AmpR	Invitrogen
p6H.Ag43.1_p_	nt181-2013 of Ag43 in-frame with pDEST17 6His coding sequence	present study
p6H.pAT_p_	nt334-1956 of pAT in-frame with pDEST17 6His coding sequence	present study

genomic DNA sequence:

a Wellcome Trust Sanger Institute, Cambridge, UK;

b jcvi (J. Craig Venter Institute).

CmR = chloramphenicol resistance; KmR = kanamycin resistance.

AmpR = ampicillin resistance.

*ccd*B = DNA gyrase toxin permits negative selection or plasmids which have not undergone *in vitro* recombination.

6His = polyhistidine tag.

nt: = nucleotides.

### *In silico* analyses

A number of parallel bioinformatics approaches were used to identify candidate AT genes in recently sequenced ETEC strains. Strains B7A and E24377A were searched for highly conserved autotransporter domains using the Pfam database including the autotransporter beta domain (http://pfam.sanger.ac.uk//family/PF03797) and the pertactin domain (http://pfam.sanger.ac.uk//family/PF03212). The resulting sequences containing these domains were used to identify additional autotransporters in the genome of ETEC strain H10407 available in un-annotated form via the Sanger Institute (http://www.sanger.ac.uk/Projects/E_coli_H10407/), which was facilitated by interrogating the available sequence using the National Microbial Pathogen Database Resource (NMPDR) [30] on the Rapid Annotation Subsystem Technology (RAST) server (http://RAST.nmpdr.org/) [31]. SignalP (http://www.cbs.dtu.dk/services/SignalP/) was used to identify potential signal peptide encoding regions of the predicted AT coding sequences. BLASTP (http://blast.ncbi.nlm.nih.gov/Blast.cgi) and Pfam domain searches of each putative AT peptide were used to define the conserved beta barrel transport domain (TIGR01414, and pfam03797, in the NCBI Conserved Domain Database http://www.ncbi.nlm.nih.gov/Structure/cdd/cdd.shtml and Pfam databases, respectively). Peptide sequence alignments of AT proteins common to the three ETEC strains used ClustalW [32] and MUSCLE [33] alignment algorithms performed locally (Mac OS 10.5.8) with an additional alignment plug-in (v1.06) for CLC Main Workbench software (v 5.5).

### Molecular cloning of autotransporter passenger domains

Regions corresponding to the majority of each passenger domain (region between the end of the putative signal peptide encoding sequence and the beginning of the beta barrel domain) were amplified using primers bearing *att*B flanking sequences by high fidelity PCR (Platinum PCR SuperMix, Invitrogen). A complete list of primers used in these studies is included in table 2. Resulting amplicons were agarose gel-purified (Ultra Clean 15, MOBIO). Amplicons containing *att*B flanking sequences were cloned by recombination with the lambda *att*P sites on the entry vector pDONR221 (BP Clonase II, Invitrogen), and transformed into DH10B One Shot *ccd*B Survival T1 Chemically Competent *E. coli* (Invitrogen). Colonies were selected and patched onto kanamycin and chloramphenicol plates. Plasmid DNA extracted from kanamycin-resistant, chloramphenicol-sensitive colonies was analyzed by restriction digest to confirm presence of the appropriate insert. pDONR221 entry clones were then recombined with pDEST17 (LR Clonase II) placing the passenger domains in-frame with an amino-terminal polyhistidine tag in the resulting expression plasmid. Cloning reactions were used to transform DH5α to ampicillin resistance. Plasmid DNA from ampicillin-resistant, chloramphenicol-sensitive colonies was analyzed first by restriction endonuclease digestion, then sequenced using T7 promoter primer 5′-TAATACGACTCACTATAGG-3′ to confirm that the 6His- and passenger encoding sequences were in-frame. Resulting plasmids were then used to transform *E. coli* expression strain BL21-A1.

**Table 2:** Primers used for amplification of AT passenger domains.

gene	forward primer5′-ggggacaagtttgtacaaaaaagcaggctgg+	reverse primer5′-ggggaccactttgtacaagaaagctgggttta+
pAT	…tatactaacgaaaccttc-3′	…gccatgacgagagttggt-3′
agn43.1	…gtggcgattgcgctgtct-3′	…tacaccggtctgatggct-3′
*eatA*	…gcaacagttaatgcagatata-3′	…ccgtaaatctcccatacg-3′

Forward primers begin with sequence containing *att*B1 sites (underlined) and additional nucleotides (gg) to place following sequence in-frame with polyhistine coding sequence in ultimate expression plasmid followed by gene-specific target sequences (…xxx-3′).

Reverse primers begin with sequence containing attB2 sites (underlined) followed by stop codon (**tta**), then gene-specific target sequence.

### Production of recombinant polyhistidine-tagged passenger proteins

To produce recombinant polyhistine-tagged passenger proteins cultures of BL21-A1 containing pDEST17-encoded 6His-passenger clones were grown overnight at 37°C in Luria broth containing ampicillin (100 µg/ml). After diluting 1∶100 in 100 ml of fresh media, cultures were grown at 37°C, 225 rpm for approximately 3 hours to an OD_600_ of approximately 0.6, then induced by the addition of L-arabinose to a final concentration of 0.02%. After approximately 2 hours, cultures were centrifuged for 5 minutes (4°C, 10,000×g), and the resulting pellet saved and frozen at −80°C for subsequent processing. Pellets were thawed on ice, and resuspended in binding buffer containing 20 mM Tris, 8 M Urea, 500 mM NaCl, 5 mM imidazole, protease inhibitor cocktail (1×, Sigma P8465) and PMSF (1 mM), at pH 8.0. After lysis on a rotator for 30 minutes, the suspension was centrifuged for 10 minutes at 10,000×g at 4° C. Recombinant polyhistidine-tagged autotransporter proteins (rATp) were then purified from clarified lysates using immobilized metal affinity chromatography (IMAC) in small-scale (His SpinTrap columns (GE Healthcare) or in larger scale by using Ni-Sepharose columns (HisTrap HP, GE Healthcare). Proteins were eluted over an imidazole gradient produced on a low-pressure chromatography system (BioLogic LP, BioRad). Purity of recombinant proteins was assessed by SDS-PAGE. Where necessary, fractions containing the protein of interest were further purified by ion exchange (HiTrap Q) or by SDS-PAGE and subsequent electroelution (Mini Whole Gel Eluter, BioRad).

### Antibody production and affinity purification

Polyclonal rabbit antisera were produced as previously described (Rockland Immunochemicals, Gilbertsville, PA). Mouse polyclonal antibodies were obtained upon sacrifice of mice following completion of intranasal immunization with either adjuvant control or adjuvant and recombinant protein of interest as described below. Antibodies were affinity-purified as previously described [13], [34]. Briefly, antibodies were absorbed onto immobilized antigen of interest on nitrocellulose, then eluted in 100 mM glycine, pH 2.5 followed by neutralization with 1 M Tris, pH 8.0.

### Immunoassays

To detect polyhistidine-tagged rATp proteins transferred onto nitrocellulose, blocking was performed for one hour in 5% skim milk in tris-buffered saline (pH 7.2) containing 0.05% Tween-20 (TBS-T), and subsequent immunoblotting used purified rabbit polyclonal anti-His serum (1∶500) and goat anti-rabbit immunoglobulin G (Fc)-Horseradish peroxidase (HRP) (1∶10,000). All wash and incubation steps were performed in Tris-Buffered Saline (pH 7.2)- 0.05% Tween 20. Detection used luminol-based chemiluminescent substrate.

Assessment of immune responses to individual rATp proteins was carried out in a similar fashion using pooled primary convalescent sera (1∶500) from mice following infection with ETEC strain H10407. Pooled, pre-immune sera (1∶500) from the same mice was used as a primary antibody control. Sera obtained from ETEC-infected children (ICDDR,B) Dhaka, Bangladesh, and uninfected (age-matched) controls (LeBonheur Children's Medical Center, Memphis, TN), were tested at a 1∶2048 dilution. Primary antibody binding was detected using an HRP-labeled secondary (anti-mouse or anti-human) antibody that detects IgA, IgM and IgG (total Ab) and developed using sensitive chemiluminescent substrate (SuperSignal West Femto, Thermo Scientific).

Kinetic ELISA assays were performed as previously described [24]. (For an in-depth discussion comparing kinetic and standard end-point ELISA techniques, the reader is referred to an earlier review available at http://www.biotek.com/resources/docs/KineticAppNoteFinal.pdf) [35]. ELISA wells were coated overnight at 4°C with (100 µl/well) of individual rATp proteins diluted to a final concentration of 4 µg/ml in 0.1 M NaHCO_3_ buffer (pH 8.6). Plates were washed three times with TBS-T, blocked with TBS-T containing 1% BSA (Blocker, Thermo Scientific) for 1 hour at 37°C. After washing briefly with TBS-T, plates were incubated with dilutions of primary antibody in TBS-T/1% BSA for 1 hour at 37°C, and again washed as above. Next, plates were incubated (1 h, 37°C) with goat anti-human or goat anti-mouse secondary antibody, which recognizes IgA, IgM, and IgG, at a final concentration of 1∶10000 in TBS-T-BSA. After washing, plates were developed with TMB (3,3′,5,5′-Tetramethylbenzidine) peroxidase substrate (Kirkgaard and Perry Laboratories). Kinetic absorbance measurements [36] were determined at 650 nm, acquired at 60 second intervals (Molecular Devices Spectramax 340PC microplate reader). SoftMax Pro v5.0.1 was used for data recording and processing and the rate of substrate development was expressed as the Vmax in milli-units/min.

### Immunization of mice

Mice were vaccinated intranasally with candidate antigens as previously described [37]. Briefly, groups of 10 ICR [38] mice received either IVX908 (Protollin®) [39] (7.5 µg) alone (controls), or IVX908 (7.5 µg) + rATp domains (20 µg) on days 0, 14, 28. Sera were collected on day 0 prior to immunization (pre-immune) and again 7–14 days after the final vaccination (post-immune). Prior to bacterial challenge (day 42), 5–6 fresh fecal pellets were collected from each mouse and immediately suspended in 1.5 ml of extraction buffer containing Tris (10 mM), NaCl (100 mM), Tween-20 (0.05%), and sodium azide (5 mM) pH 7.4.

### Intestinal colonization studies

Approximately 1 week after completion of the immunization schedule, mice were challenged with jf876, a derivative of ETEC H10407 containing a KmR marker in the *lacZ* gene as previously described [37], [40]. Two independent challenge studies were completed; each involved a total of 30 mice including 10 controls (IVX908 only), and groups of 10 mice vaccinated with either of the two rATp proteins. Briefly, mice were treated with streptomycin (5 g/liter) in their drinking water to eliminate colonization resistance from competing normal enteric flora. Food was then withheld 12 hours prior to ETEC challenge and sterile water was used to replace the streptomycin solution. All mice received cimetidine (50 mg/kg via intraperitoneal injection) 2 hours prior to administration of bacteria. Mice were then challenged by gavage administration of bacterial strain jf876 with total dose between 10^4^ and 10^5^ cfu/mouse. Previous studies have demonstrated that at 24 hours after inoculation with ETEC in the murine model, the number of colonizing colony forming units in the small intestine parallel values obtained at later time points (72 hours) [38]. Therefore, in these studies, approximately 24 hours after challenge mice were sacrificed, samples of ileum were harvested, then solubilized in saponin solution to release bacteria, and finally plated onto Luria agar plates containing kanamycin (25 µg/ml). Colonies were counted after incubation overnight at 37°C. Intestinal lysates with no bacteria recovered were assigned a value of 1 cfu (the lower limit of detection) as previously described [37]. Care was taken to minimize the use of animals in these experiments where possible. When necessary, appropriate steps were taken to ameliorate suffering of mice during vaccination and testing. The Institutional Animal Care and Use Committees of the University of Tennessee Health Sciences Center, and the VA Medical Center approved studies presented here. All procedures involving mice complied with Public Health Service guidelines, and The Guide for the Care and Use of Laboratory Animals.

### Flow cytometry

To evaluate surface expression of autotransporter passenger domains, suspensions of H10407 in phosphate buffered saline (PBS, pH 7.2) were first fixed with 2% paraformaldehyde for 15 minutes. After washing twice with PBS, cells were blocked with 1% BSA in PBS for 30 minutes. The resulting cell suspension was incubated with either pre- or post-immune mouse sera (diluted 1∶50 in blocking buffer) for 1 hour at room temperature (RT). After washing three times with PBS, cells were incubated with Alexa-Fluor (488)-labeled anti-mouse IgG [1∶250] for 1 hour at RT, washed three times, and resuspended in PBS. Analysis of cell-bound fluorescence by flow cytometry used a BD FACSCalibur 4-color, dual-laser flow cytometer equipped with a FACStation data management system.

## Results

### Identification of additional ETEC autotransporter genes

**Figure 1:**
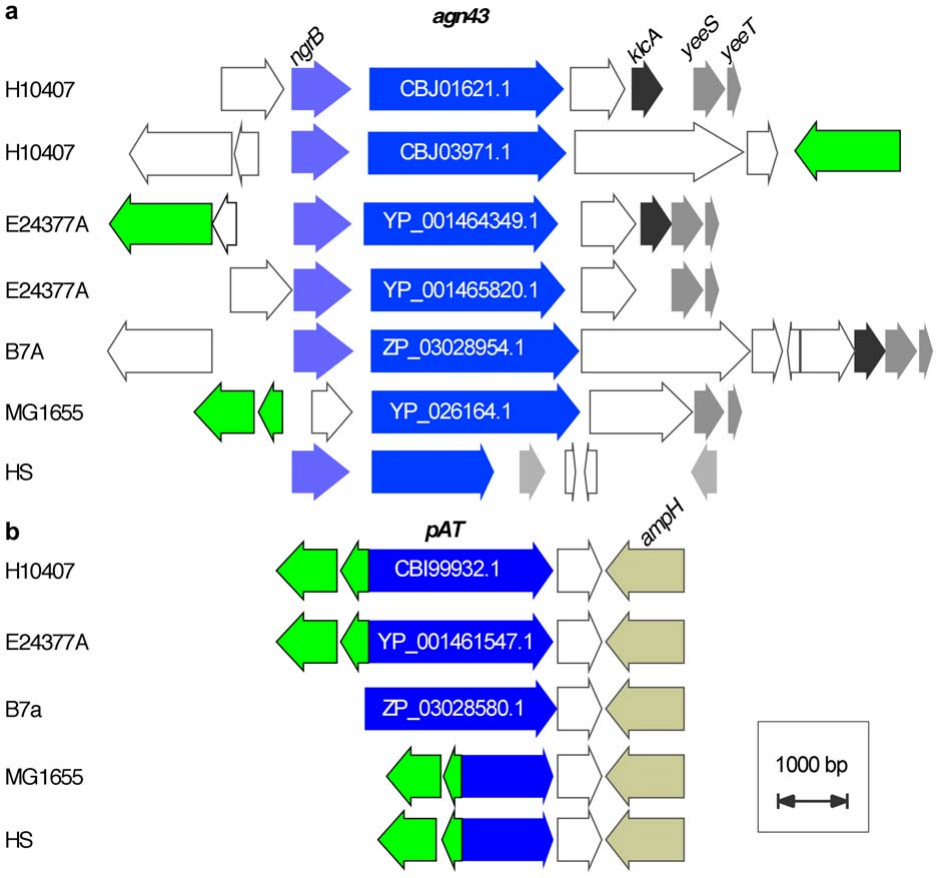
Chromosomal context of conserved autotransporter genes in ETEC and nonpathogenic *E. coli genomes*. **a.** location of antigen 43 (*agn*43) genes in the chromosomes of ETEC strains (H10407, E24377A, and B7A) and nonpathogenic *E. coli* strains (MG1655, and HS). Genes are shaded by similarity. Individual autotransporter genes are depicted in blue. Putative full-length autotransporter genes include their assigned ncbi protein reference numbers. Mobility elements are depicted in green. Open arrows represent hypothetical genes. **b.** location of *pAT genes* (in blue). Figures are based on RAST annotations (http://rast.nmpdr.org/).

*In silico* analysis of available ETEC genomes using searches for one or more of the known highly conserved AT domains identified multiple autotransporters in the genome of each ETEC strain. From these, we selected AT genes that were present in ETEC, but absent in the recently sequenced *E. coli* HS commensal isolate [41], for additional study (table 3). All three genomes shared at least one copy of both antigen43, and the pAT autotransporter genes (figure 1). Similar to other pathovars [42], we identified two copies of the *agn*43 gene in both H10407, and E24377A, but a single copy in B7A. The amino acid sequence of the passenger domains of these Ag43 molecules was highly conserved (supporting figure S1a), while the pAT passenger domains exhibited approximately 70% identity and 80% similarity (supporting figure S1b).

**Table 3:** Comparison of selected autotransporter genes in ETEC and nonpathogenic *E. coli strains*.

Strain	RAST*	ncbi	UniProt	name	length (AA)	identical(%)	similar(%)	score^c^
genes similar to antigen 43								
H10407	2318	CBJ01621.1	E3PBK1	*agn*43.2^a^	973	-	-	-
	4884	CBJ03971.1	E3PD73	*agn*43.1^b^	989	93	96	1762
E24377A	1041	YP_001464349.1	A7ZRC0	*agn*43.2^a^	948	96	97	1817
	2475	YP_001465820.1	A7ZVJ1	*agn*43.1^b^	948	96	97	1815
B7A†	2219	ZP_03028954.1	B3HDS9	*agn*43	1039	74	79	1391
MG1655	2060	YP_026164.1	P39180	agn43	1039	71	76	1359
HS	2001	YP_001458811.1	A8A1M4	*agn*43**′**	608	95	97	1042
genes similar to *pAT*								
H10407	459	CBI99932.1	E3PFJ1	*pAT*	917	-	-	-
E24377A	2943	YP_001461547.1	A7ZIB8	*pAT*	922	82	87	1337
B7A	1772	ZP_03028580.1	B3HCJ6	*pAT*	962	75	83	1330
MG1655	379	AAB18097.1	na	**‘**pAT	465	98	99	908
HS	407	YP_001457207.1	A7ZX20	**‘**pAT	465	93	97	810

*NMPDR RAST (Rapid Annotation using Subsystem Technology) at http://rast.nmpdr.org/.

**†:** [B7A sequence is in draft form (198 contigs), therefore locations in B7A are inferred from surrounding genes].

a alternatively, also referred to as *flu*2 for “fluffing protein” 2.

b alternatively, also referred to as *flu*1 for “fluffing protein” 1.

c by BLASTP algorithm http://blast.ncbi.nlm.nih.gov/Blast.cgi?CMD=Web&PAGE_TYPE=BlastHome.

na: not available.

**‘**denotes truncated pseudogene.

Although genes encoding proteins similar to pAT were found in the *Enterobacteriaceae* by BLAST searches of the prototype pAT molecule from H10407 (Uniprot designation E3PFJ1) in the UniprotKB database, and were widely distributed among various pathovars of *E. coli* as well as *Salmonella, and Shigella species* (taxonomic distribution of these proteins is included in supporting figure S2), only those in *E. coli* exhibited more than 80% identity. As demonstrated in table 4, pAT homologues were identified in other diarrheagenic *E. coli* pathotypes including enterohemorrhagic strains (EHEC), enteropathogenic isolates (EPEC), and uropathogenic *E. coli* (UPEC), and extraintestinal pathogenic *E. coli* (ExPEC) isolated from meningitis. However, we also identified potential pAT homologues in non-HS commensal isolates from humans [43] as well as animals [44], suggesting that while pAT may not be essential for commensalism, it is not unique to pathogenic strains.

**Table 4:** pAT-like autotransporters in other *E. coli* pathovars and commensal *E. coli* strains^1^^,^ 2.

accession(UniProtKB)	strain	pathotype	origin; clinical features	length	identity (%)	reference
C8U260	12009	EHEC	Isolated in Japan in 2001 from a patient with a sporadic case of bloody stool	992	89	[74]
E7HXI7	E128010	diarrheagenic	Bangladesh, infant diarrhea	945	89	[75]
B3WPL7	B171	EPEC	infant-toddler daycare diarrheal outbreak	992	89	[76]
B3I9D8	E22	EPEC	weaned-rabbit diarrhea	992	89	[77]
D2ND18	SE15	commensal	isolated from health adult, Japan	995	83	[43]
F5MCM6	AA86	commensal	healthy cow feces	995	86	[44]
E1P987	ABU83972	-	asymptomatic bacteriuria	995	83	[78]
B3HW28	F11	UPEC	cystitis	995	83	[79]
Q1RFH4	UTI89	UPEC	cystitis	995	83	[80]
D5CVR9	IHE3034	ExPEC	meningitis	995	83	[81]

notes:

1 recorded in the table are results obtained after BLASTP searches of the UniProt database with pAT from H10407, [http://www.uniprot.org/uniprot/E3PFJ1] using following parameters: database was UniProtKB; threshold = 0.0001; matrix = auto; low complexity regions filtered; gaps permitted.

2 (the top ten BLAST hits with established disease association metadata are included).

### Surface expression of autotransporter passenger domains

**Figure 2:**
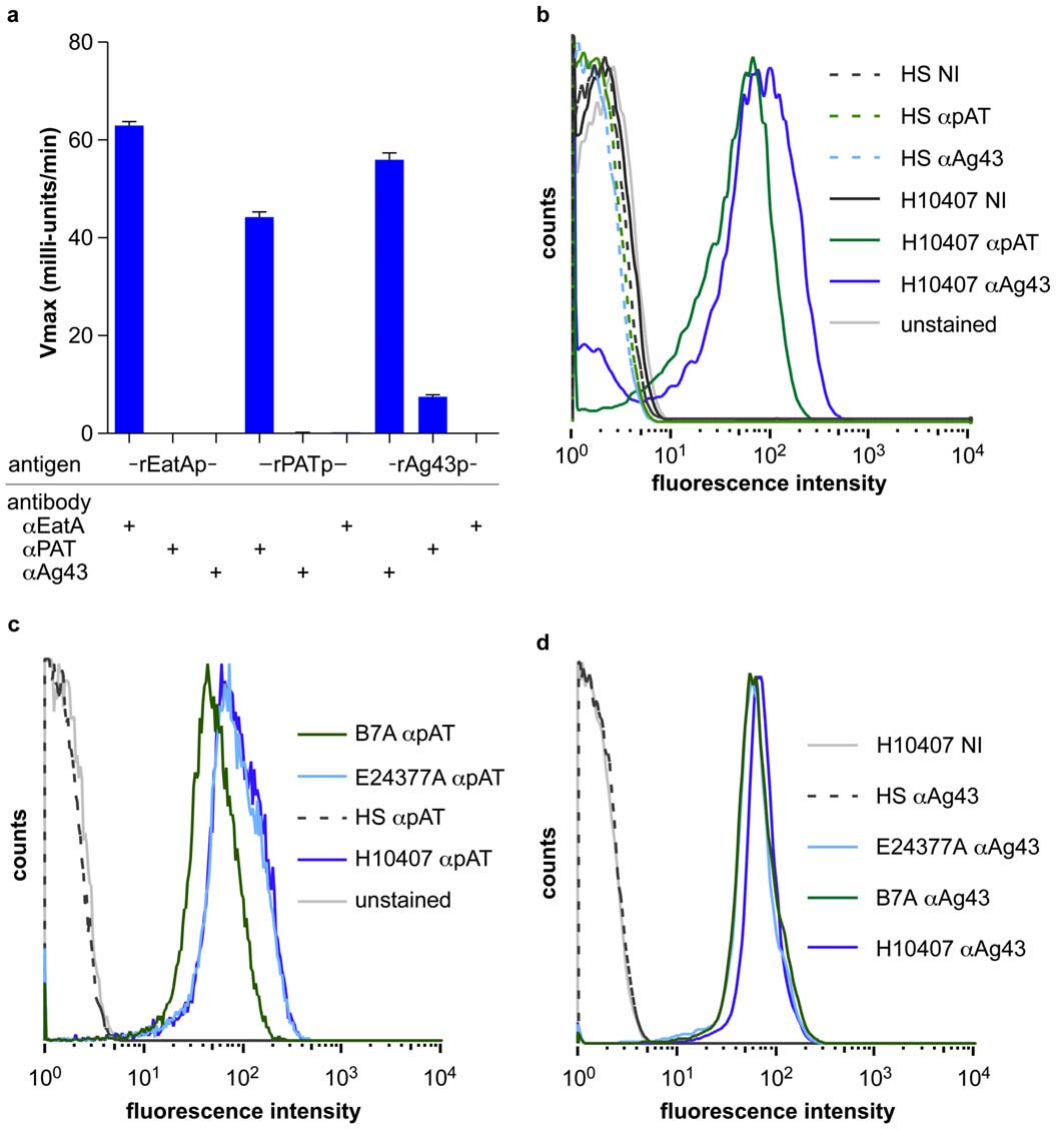
Surface expression of autotransporter proteins by ETEC. **a.** specificity of antibodies directed against ETEC autotransporter passenger domains. Data shown are kinetic ELISA data obtained at a 1∶1024 dilution of all primary antisera. **b.** flow cytometry study comparing the presence of pAT and Ag43 AT passenger domains on the surface of ETEC strain H10407 compared to the HS *E. coli* commensal strain. Pre-immune (NI for non-immune) sera, as well as unstained bacteria (no primary antibody) are shown as controls **c.** Examination of pAT surface expression by ETEC strains B7A and E24377A relative to the H10407 (+ control) and the commensal HS (negative control); unstained control used no primary antibody. **d.** Examination of Ag43 surface expression by ETEC strains B7A and E24377A relative to the H10407 and HS controls. Flow cytometry histograms depict intensity of fluorescence signal on the abscissa (x-axis) while the relative frequency of bacteria counted is depicted on the ordinate (y-axis).

Passenger domains of autotransporter proteins are exported to the bacterial surface where they may be cleaved and appear in the supernatant as in the case of EatA [14], or remain largely associated with bacterial cell surface as has been described for antigen 43 [42]. Antibodies raised against the recombinant passenger domains of Ag43, pAT, and EatA were tested in kinetic ELISA assays against the corresponding antigen as well as each of the other rATp domains. These studies revealed little or no cross-reactivity between antigens, suggesting that these proteins are immunologically distinct (figure 2a). Flow cytometry detected significant amounts of passenger domains of Ag43 and pAT associated with the bacterial cell surface of ETEC H10407, but not with the HS commensal strain (figure 2b). Likewise, pAT (figure 2c) and Ag43 (figure 2d) were identified on the surface of both the E24377A, and B7A ETEC strains.

### Passenger domains of conserved chromosomally-encoded autotransporter proteins are recognized during infection

Because passenger domains of autotransporter proteins are exported to the bacterial surface, they often elicit an immunologic response in the host during the course of infection [45], [46], [47]. Furthermore, recent immuno-proteomic studies have indicated that autotransporters are recognized during the course of experimental infection in mice [26]. Therefore, we examined the immune responses to conserved, chromosomally encoded autotransporter proteins identified in ETEC, using sera obtained from mice following experimental infection and from humans following natural ETEC infections.

**Figure 3:**
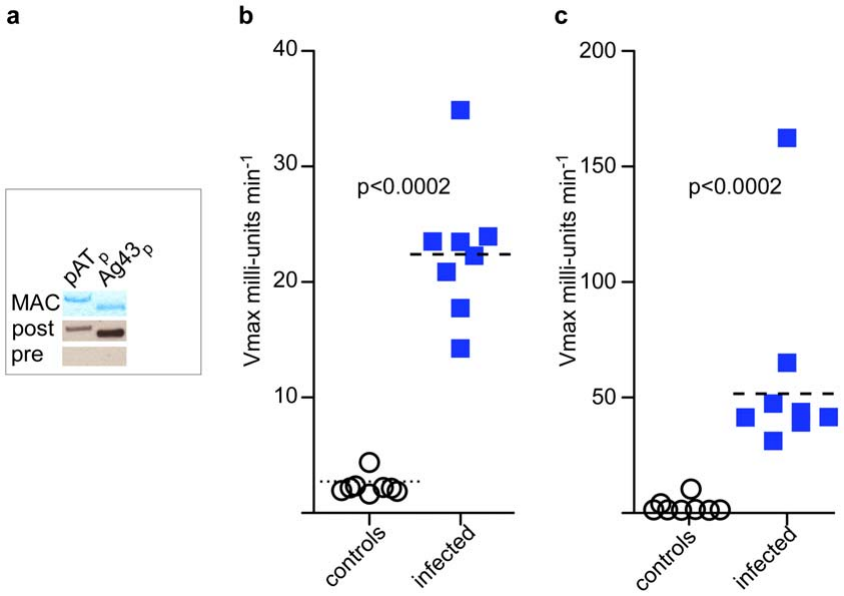
Immunogenicity of autotransporter protein passenger domains. **a.** passenger domains of autotransporters pAT and Ag43 are recognized by during the course of experimental murine infection with ETEC H10407. Shown are metal affinity chromatography-purified antigens (MAC) used in analysis, followed by corresponding immunoblots using pooled sera obtained from mice before and after intestinal challenge with ETEC H10407. **b.** human ETEC convalescent sera (from patients infected with ETEC obtained at ICDDR,B), but not age-matched sera from uninfected controls (from LeBonheur Children's Hospital, Memphis) recognize the ETEC H10407 autotransporter passenger domain of pAT (RAST designation 459). **c.** human ETEC convalescent sera, but not age-matched control sera recognize passenger domain of the ETEC H10407 autotransporter Ag43 (RAST designation 2318).

Immunoblotting studies of both of the H10407 autotransporters tested H10407_Ag43, and H10407_pAT, demonstrated that the passenger domain of both of these antigens is recognized during the course of experimental intestinal infection in mice (figure 3 a). This was also true of TibA and EatA passenger domains (not shown) as predicted by earlier proteomic studies, however we chose to focus here on the conserved, chromosomally encoded antigens, Ag43 and pAT. Subsequent studies demonstrated that both Ag43 and pAT are recognized during the course of human infections with ETEC (figure 3 b, c).

### Immunization with passenger proteins protects against ETEC colonization

**Figure 4:**
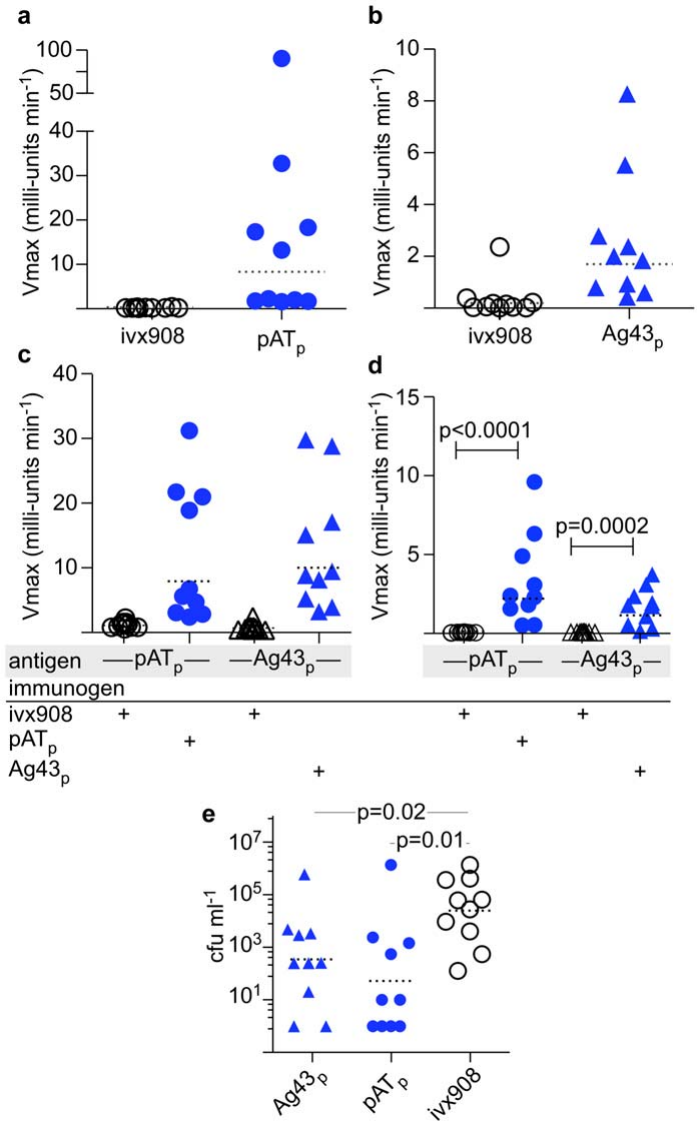
Vaccination with autotransporter passenger domains protects against intestinal colonization with ETEC in a murine model. **a.** kinetic ELISA data showing serologic responses of animals vaccinated with passenger domain of autotransporter designated pAT_p_ (closed circles) relative to adjuvant-only controls (ivx908, open circles). **b.** kinetic ELISA data for serologic responses of animals immunized with Ag43 passenger (Ag43_p_, closed triangles) relative to ivx908-only controls. **c.** kinetic ELISA of fecal antibodies (total IgG, IgM, IgA) obtained from pATp and Ag43p immunized mice (closed symbols) relative to adjuvant-only controls (open symbols); antigen (in shaded region) on the x-axis refers to the antigen used to coat ELISA wells. **d.** kinetic ELISA of fecal IgA antibody following vaccination with either the pAT or Ag43 passenger domains (closed symbols) relative to adjuvant-only controls (ivx908, open symbols) **e.** KmR-bacteria recovered from intestinal lysates following challenge with (1.2×10^4^ cfu/mouse) of jf876 (ΔlacZYA::KmR mutant of ETEC strain H10407). Dashed horizontal lines reflect geometric means. All statistical comparisons were performed using two-tailed Mann Whitney analysis.

To explore the utility of ETEC autotransporter proteins as vaccine candidates, we examined both the immunogenicity and protective efficacy of individual passenger domains for Ag43 and pAT in a murine model of ETEC infection. For these studies, we used the mucosal adjuvant Protollin (ivx908), a mixture of *Shigella flexneri* 2a LPS, and meningococcal outer membrane proteins. When delivered intranasally, Protollin (IVX908) elicits high levels of S. flexneri LPS-specific fecal IgA [48], and when combined as an adjuvant with other proteins, it promotes similarly robust immune responses to target antigens [39], [49]. Immunization with either of the passenger domains for antigen 43 or pAT resulted in significant increases in total serum and fecal antibody levels in immunized mice relative to adjuvant-only (ivx908) controls (figure 4a–c). Importantly, immunization with either rPATp or rAg43p resulted in significant increases in fecal IgA directed at the respective antigens (figure 4d).

Immunization with either the antigen43 or pAT recombinant passenger domains provided significant protection against subsequent colonization with ETEC relative to adjuvant-immunized controls (figure 4e).

## Discussion

Despite the global importance of enterotoxigenic *E. coli* infections, there is presently no vaccine against these pathogens that would offer sustained, broad-based protection [50]. Vaccine development for ETEC is a challenge for a number of reasons. First, the inherent plasticity of *E. coli* genomes makes discovery of conserved, pathotype-specific antigens difficult [51], [52]. In addition, much of the ETEC vaccine development effort to date has focused on the plasmid-encoded colonization factors (CFs). Unfortunately, antigenic heterogeneity and lack of appreciable cross protection between CFs have been impediments to this approach. To date, over twenty-five different CFs have been identified in ETEC [53], and many strains do not appear to make any of the known antigens [9], [54]. Finally, carefully conducted epidemiologic studies of natural ETEC infections have suggested that LT and perhaps other as yet unidentified chromosomally-encoded antigens [29], in addition to the plasmid-encoded CFs, could be involved in protective immune responses.

Because we have recently demonstrated that several autotransporter proteins are recognized following experimental and natural ETEC infections [26], we chose to investigate the possible contribution of conserved chromosomally-encoded autotransporter proteins. The studies here suggest that the passenger domains of these autotransporters are recognized during the course of both experimental infections in animals and naturally-occurring infection in humans, and they validate recent immunoproteomic data obtained with the prototype H10407 ETEC strain using sera from infected mice or human convalescent sera [26].

Two additional autotransporters have previously been examined in ETEC pathogenesis. These include the chromosomally-encoded TibA adhesin [55], [56] protein and, EatA [14], a plasmid-encoded member of the SPATE family (serine protease autotransporters of the *Enterobacteriaceae*). Both proteins are recognized during the course of experimental and naturally occurring infections [26]. Interestingly, the EatA protein appears modulate adhesion and colonization by digesting another recently described virulence molecule, the EtpA two-partner secretion exoprotein, an adhesin [12], [13]. In turn, this modulation of adherence appears to be required for optimal delivery of heat-labile toxin (LT), a critical ETEC virulence molecule [57]. Although recent data suggest that both EtpA and EatA are reasonably conserved within the ETEC pathovar [52], [58], [59], the inherent plasticity of *E. coli* genomes, and the relative paucity of pathovar-specific virulence genes [51] identified to date suggests that additional effort is warranted to explore the potential utility of other highly conserved surface structures as vaccine candidates.

Although it is likely that autotransporters contribute the overall fitness of ETEC as a pathogen, neither of the proteins under study here has been shown to contribute to the pathogenesis of ETEC. Antigen 43 does however appear to be of importance in the pathogenesis of other *E. coli* pathovars, including uropathogenic *E. coli* (UPEC). In the urinary tract, Ag43 is expressed in intracellular biofilm-like pods [60], and particular variants appear to contribute to biofilm formation, and colonization of the urinary tract [61]. Similarly, immunoproteomic studies demonstrate that this antigen is also expressed [62] and recognized [63] during the course of *E coli* urinary tract infections in humans.

Interestingly, in a study of an *E. coli* laboratory isolate, Ag43 contributed to biofilm formation *in vitro*, but did not appear to play a role in intestinal colonization in a murine model [64]. Nevertheless, some studies have suggested that specific Ag43 alleles segregate with diarrheagenic *E. coli* pathogens compared to other isolates from other pathovars [65], and that in general Ag43 was more commonly found in pathogens than in commensal strains.

Assessing the precise contribution of given antigens to the protective immune responses that develop following infection, or even following vaccination can be challenging [66], [67]. While serologic responses to some CFs such as CFA/I have previously been correlated with a protective immune response to ETEC [28], it is likely that protection seen following natural infections reflects a composite response to a number of antigens.

Additional studies will be needed to determine the utility of these antigens as well as other autotransporters in ETEC vaccines. The surface expression of the autotransporter passenger domains, their immunogenicity, and preliminary data presented here support the concept that this class of molecules could serve as protective antigens. Although the inherent plasticity of *E. coli* genomes [68] in general poses an impediment to vaccine development for ETEC, important data emerging from the DNA sequencing of multiple ETEC genomes does suggest that these pathogens maintain a core subset of relatively pathovar-specific genes, such as the *eatA* autotransporter gene, that might serve as suitable targets [52], [59]. The suggestion that relatively few genes separate the ETEC pathovar from commensal *E. coli* [58] is an important consideration in moving forward with putative ETEC vaccines. The data presented here suggest that other autotransporters not unique to the ETEC pathovar contribute to intestinal colonization, a critical step in ETEC pathogenesis as well as the host immune response to these important pathogens. Whether the more widely distributed ATs such as Ag43 and pAT are truly dispensable for the commensal *E. coli* similar to the HS prototype strain will be an important consideration in designing both subunit and live-attenuated vaccine strategies.

## Supporting Information

Figure S1 **Conservation of antigen 43 and pAT passenger domains.** Shown are MUSCLE alignments for predicted AT passenger regions selected from three sequenced ETEC strains, (H10407, E24377A, and B7A): **a.** Ag43 autotransporters H10407_Ag43.1, H10407_Ag43.2, E24377A_Ag43.1, and E24377A_Ag43.2 and B7A_Ag43. (arrowhead shows predicted signal peptide cleavage site) **b.** pAT from H10407, E24377A, and B7A.
(PDF)

Figure S2 **Distribution of potential pAT-like proteins in the** ***Enterobacteriaceae*** **.** The distribution of potential pAT-like proteins was defined by BLASTP searches of Enterobacteriaceae with pAT from H10407 [http://www.uniprot.org/uniprot/E3PFJ1] using the UniProtKB database (threshold E value of 0.0001; filtered for low regions of complexity). The degree of identity varied from 89% (for enterohemorrhagic *E. coli* strain 12009) to less than 50% for the other *Enterobacteriaceae*. Only *E. coli* proteins were more than 80% identical to pAT. The closest homologues in *E. coli* are shown in table 4.
(TIF)
