# Supplementary material for: Directed Evaluation of Enterotoxigenic Escherichia coli Autotransporter Proteins as Putative Vaccine Candidates
Source: PLoS Negl Trop Dis. 2011 Dec 6;5(12):e1428. doi: 10.1371/journal.pntd.0001428 (PMC3232201; doi:10.1371/journal.pntd.0001428)
Supplement: Figure S1 — Conservation of antigen 43 and pAT passenger domains. Shown are MUSCLE alignments for predicted AT passenger regions selected from three sequenced ETEC strains, (H10407, E24377A, and B7A): a. Ag43 autotransporters H10407_Ag43.1, H10407_Ag43.2, E24377A_Ag43.1, and E24377A_Ag43.2 and B7A_Ag43. (arrowhead shows predicted signal peptide cleavage site) b. pAT from H10407, E24377A, and B7A. (PDF) [file pntd.0001428.s001.pdf]

a.

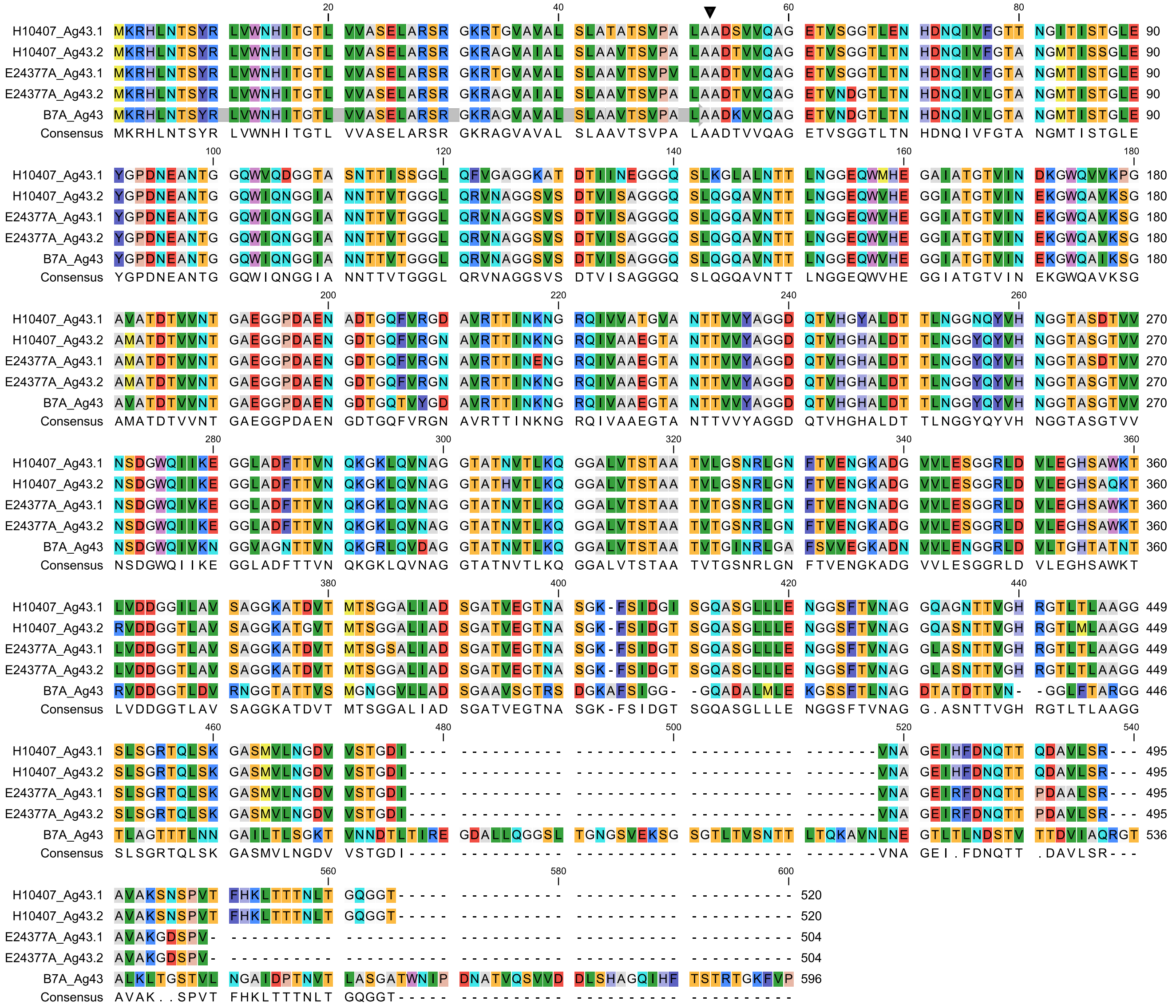
H10407_Ag43.1
H10407_Ag43.2
E24377A_Ag43.1
E24377A_Ag43.2
B7A_Ag43
Consensus

b.

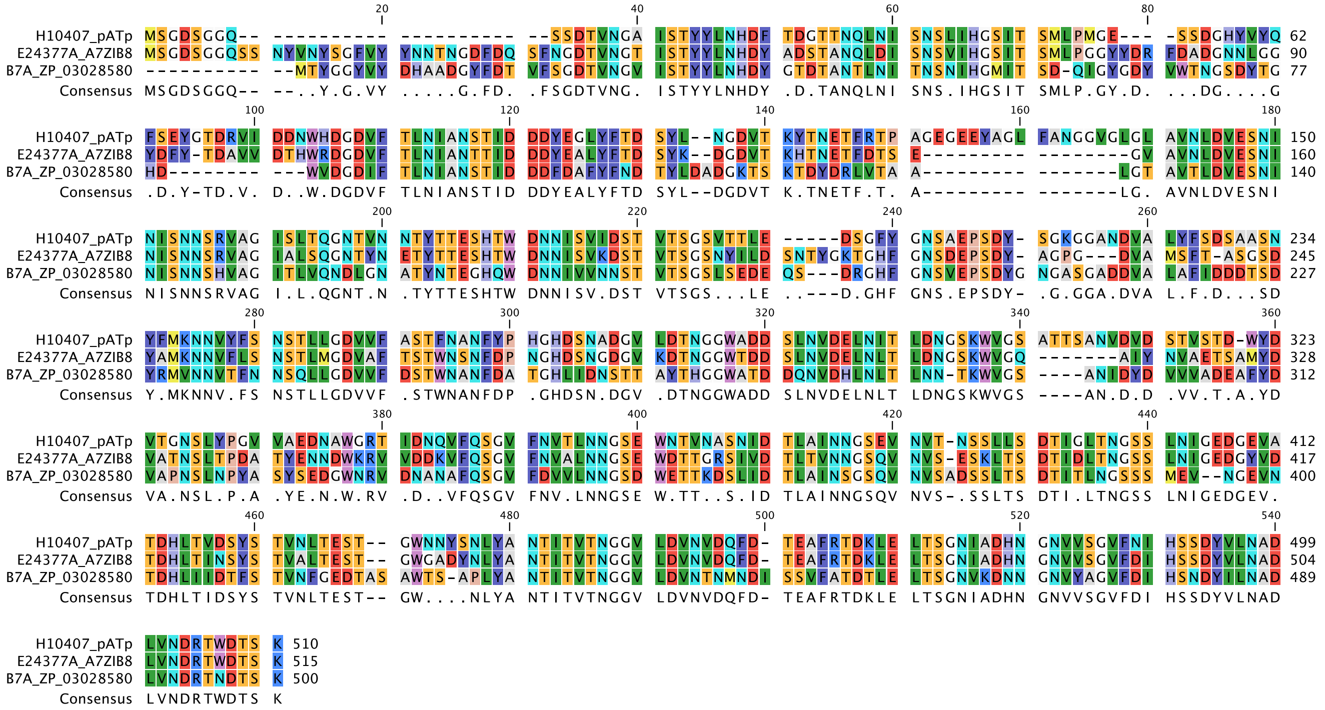
H10407_pATp
E24377A_A7ZIB8
B7A_ZP_03028580
Consensus
