# Supplementary figures and images for: Directed Evaluation of Enterotoxigenic Escherichia coli Autotransporter Proteins as Putative Vaccine Candidates
Source: PLoS Negl Trop Dis. 2011 Dec 6;5(12):e1428. doi: 10.1371/journal.pntd.0001428 (PMC3232201; doi:10.1371/journal.pntd.0001428)

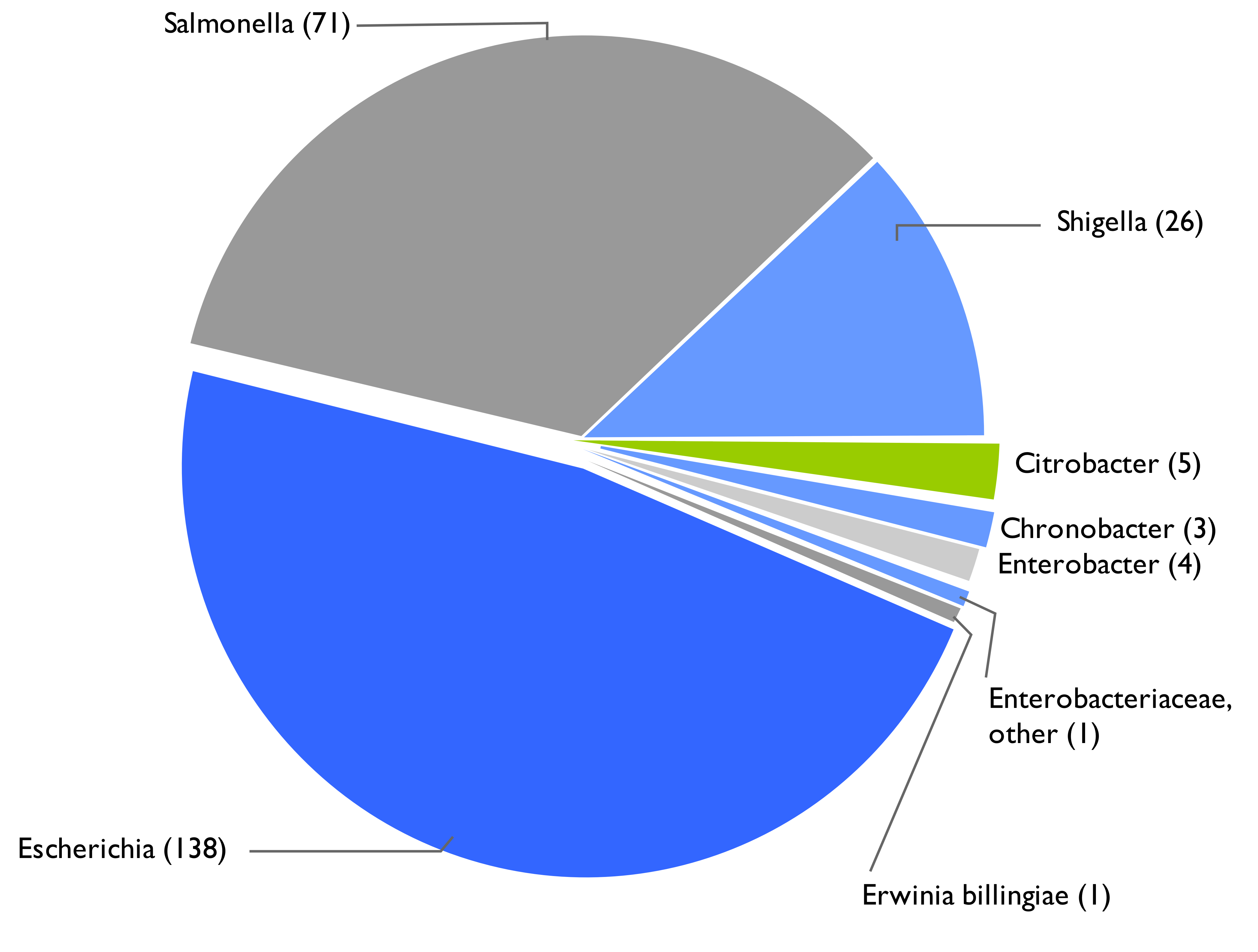

Supplement: Figure S2 — Distribution of potential pAT-like proteins in the Enterobacteriaceae . The distribution of potential pAT-like proteins was defined by BLASTP searches of Enterobacteriaceae with pAT from H10407 [http://www.uniprot.org/uniprot/E3PFJ1] using the UniProtKB database (threshold E value of 0.0001; filtered for low regions of complexity). The degree of identity varied from 89% (for enterohemorrhagic E. coli strain 12009) to less than 50% for the other Enterobacteriaceae. Only E. coli proteins were more than 80% identical to pAT. The closest homologues in E. coli are shown in table 4. (TIF) [file pntd.0001428.s002.tif]
